# Stress-Dependent Pore Deformation Effects on Multiphase Flow Properties of Porous Media

**DOI:** 10.1038/s41598-019-51263-0

**Published:** 2019-10-18

**Authors:** Amir H. Haghi*, Richard Chalaturnyk, Stephen Talman

**Affiliations:** Reservoir Geomechanics Research Group, University of Alberta, Edmonton, T6G 1H9 Canada

**Keywords:** Hydrology, Solid Earth sciences, Energy science and technology, Engineering

## Abstract

Relative permeability and capillary pressure are the governing parameters that characterize multiphase fluid flow in porous media for diverse natural and industrial applications, including surface water infiltration into the ground, CO_2_ sequestration, and hydrocarbon enhanced recovery. Although the drastic effects of deformation of porous media on single-phase fluid flow have been well established, the stress dependency of flow in multiphase systems is not yet fully explored. Here, stress-dependent relative permeability and capillary pressure are studied in a water-wet carbonate specimen both analytically using fractal and poroelasticity theory and experimentally on the micro-scale and macro-scales by means of X-ray computed micro-tomography and isothermal isotropic triaxial core flooding cell, respectively. Our core flooding program using water/N_2_ phases shows a systematic decrease in the irreducible water saturation and gas relative permeability in response to an increase in effective stress. Intuitively, a leftward shift of the intersection point of water/gas relative permeability curves is interpreted as an increased affinity of the rock to the gas phase. Using a micro-scale proxy model, we identify a leftward shift in pore size distribution and closure of micro-channels to be responsible for the abovementioned observations. These findings prove the crucial impact of effective stress-induced pore deformation on multiphase flow properties of rock, which are missing from the current characterizations of multiphase flow mechanisms in porous media.

## Introduction

An accurate representation of the variation in relative permeability and capillary pressure with phase saturation is a crucial requirement for understanding multiphase fluid flow in hydrocarbon/geothermal reservoirs^1–4^, vadose zone water transport^5^, and carbon dioxide storage projects^6,7^. Many researchers have studied the multiphase flow properties of porous media experimentally at both pore-scale and core-scales with a focus on fluid-fluid and fluid-solid interfacial interactions^8–17^. Additionally, several empirical models have been developed to fit curves on the experimental data to reproduce multiphase flow properties^18–23^. Recent analytical developments on this front have led researchers to suggest a fractal description of pore sizes in porous rocks^24^. The investigation of flows in porous media is also an important research field within the numerical community. Complex flows in porous media across scales and in the presence of catalytic or other chemical reactions have been recently numerically performed in^25–28^ wherein the authors presented novel and efficient numerical approaches to study flows in fractured media. Although diverse in methodology and material type, these experimental, analytical, and numerical studies have routinely treated the porous media as a stress-independent solid with zero solid-solid mechanical interaction. This restriction in the theoretical treatment persists despite the limited, but increasing, body of experimental^29–34^ and analytical^35–37^ evidence of changes to multiphase flow properties with effective stress-induced deformation. Complicated physical behaviors and contradictory findings continue to challenge our understanding of stress-dependent multiphase flow at pore-scale and core-scale^36,37^.

Fluid production or injection in subsurface porous media will locally change pore pressures and *in-situ* stresses^38–40^. These stress changes will, in turn, lead to pore deformation in response to the pore pressure/stress coupling effect, for which we use the term “geomechanics” to generally describe this process. The earliest theory to account this coupling effect of soils was introduced by Terzaghi^41^. However, the linear theory of poroelasticity was first borne from the pioneering work of Biot^42^ and is broadly understood from core-scale and pore-scale experimental studies^43–45^. Several models have been presented to relate relative permeability and capillary pressure curves to the pore size distribution in porous media^18–20^. Typically, few parameters are required to model these curves; these include the irreducible wetting phase (*Sw*_*ir*_) and/or residual non-wetting phase (*Snw*_*r*_) saturations, end-point relative permeability (*k*_*r−max*_), and one more parameter (*λ*), which defines any curvature in the dependency of relative permeability on the pore size distribution^19^. A further enhancement of the model was achieved by treating the pore space as a bundle of capillary tubes using fractal (*D*_*f*_) and tortuosity fractal (*D*_*t*_) dimensional parameters^46–50^. The idea of fractal distribution of capillary tubes was then used to solve classic relative permeability and capillary pressure equations^24^.

Despite an abundance of experimental studies of relative permeability and capillary pressure from both pore-scale and core-scale studies, the impact of pore deformation induced by effective stress changes on multiphase flow properties remains poorly explored^33^. For conditions of increasing effective stress, existing experimental evidences^29,32^ reveal contradictory shifts in the relative permeability curves. Recognition of this contradiction has resulted in several authors conducting experiments to tackle the challenges in core-scale experiments^33,34^ and pore-scale physical models^44^, independently. However, a comprehensive study of multiphase flow properties (e.g. relative permeability and capillary pressure) in both core-scale and pore-scale under a wide range of effective stress conditions has yet to be undertaken.

In this study, we use X-ray computed micro-tomography to quantify the structure and shape of the pores, together with the pore size distribution and two metrics (fractal dimension and degree of anisotropy), of an initially water-wet Indiana limestone specimen at atmospheric pressure. Then, through a series of core-flooding experiments using water/N_2_ pair phases, the stress-dependent porosity, absolute permeability, relative permeability, and capillary pressure of the same specimen are measured under triaxial isotropic effective stress (10 MPa to 30 MPa) and isothermal (40 °C) conditions. Using the measured stress-dependent porosity and pore strain of the core, we reconstruct 3D pore-scale proxy models of the sample at 10, 20, and 30 MPa effective stress conditions. This approach provides a simultaneous investigation of the pore and/or throat shape alteration and pore size distribution indices (e.g. fractal dimension) under variable stress conditions. Additionally, an analytical model is developed based on the fractal theory to reproduce and interpolate the stress-dependent relative permeability and capillary pressure curves. We find that increasing the effective stress results in a leftward shift in pore size distribution, closure of micro-channels, and modification to the flow path tortuosity. These changes are responsible for a dramatic upward shift in capillary pressure curve and a decrease in gas relative permeability. Executing experiments under raising effective stress conditions and constant gas flow rates, we show that increasing applied capillary pressure at the pore-scale due to an increase in gas pressure provides additional energy for the gas phase to invade smaller pore channels, leading to a subsequent decrease in the irreducible water saturation. These findings emphasize the complexity of the physical processes responsible for geomechanical controls on multiphase flow properties in porous media. Based on this research, we discuss the significant role of the stress-dependent relative permeability, capillary pressure, and irreducible wetting phase saturation for some interesting applications in engineering and natural process.

## Stress-Dependent Flow Properties from Core-Scale Experiments

A set of single-phase (water) and two-phase (water/N_2_) core flooding experiments were conducted on a water-wet Indiana limestone core in a high-pressure triaxial cell under a range of isotropic effective confining stress conditions from 0 to 30 MPa and a constant temperature at 40 °C. The equipment^34^ allowed for accurate measurement of porosity, absolute permeability, relative permeability, and capillary pressure at several different effective confining stress conditions.

First, the confined carbonate core inside the cell was fully saturated with deionized water using the procedure given in the *Materials and Methods*. Then, the required confining stress inside the cell was applied and the sample porosity measured. At each stress condition, we performed single-phase water flooding tests at several fixed rates (*q*_*w*_ = 1–5 *mL*/*min*) to obtain stress-dependent absolute permeability. Afterward, the Hassler relative permeability method^51,52^, in which water and N_2_ are simultaneously injected directly into the top of the core through two separate lines, was used to evaluate the steady-state pressure drop across the sample for each phase. These measurements were made at several water fractional flow values (*f*_*w*_ = 1, 0.8, 0.6, 0.4, 0.2, 0). For the capillary pressure measurement, we employed the modified stationary liquid method^53^, keeping the wetting phase (water) immobile in the core while imposing several steady-state gas flow rates (*q*_*g*_ = 1–5 and 8 *mL*/*min*). Subtracting the core outlet pressure at steady-state condition from the gas pressure at the inlet provided an estimate of applied dynamic capillary pressure^54^.

## Pore-Scale Observations and Conceptual Proxy Modelling

**Figure 1:**
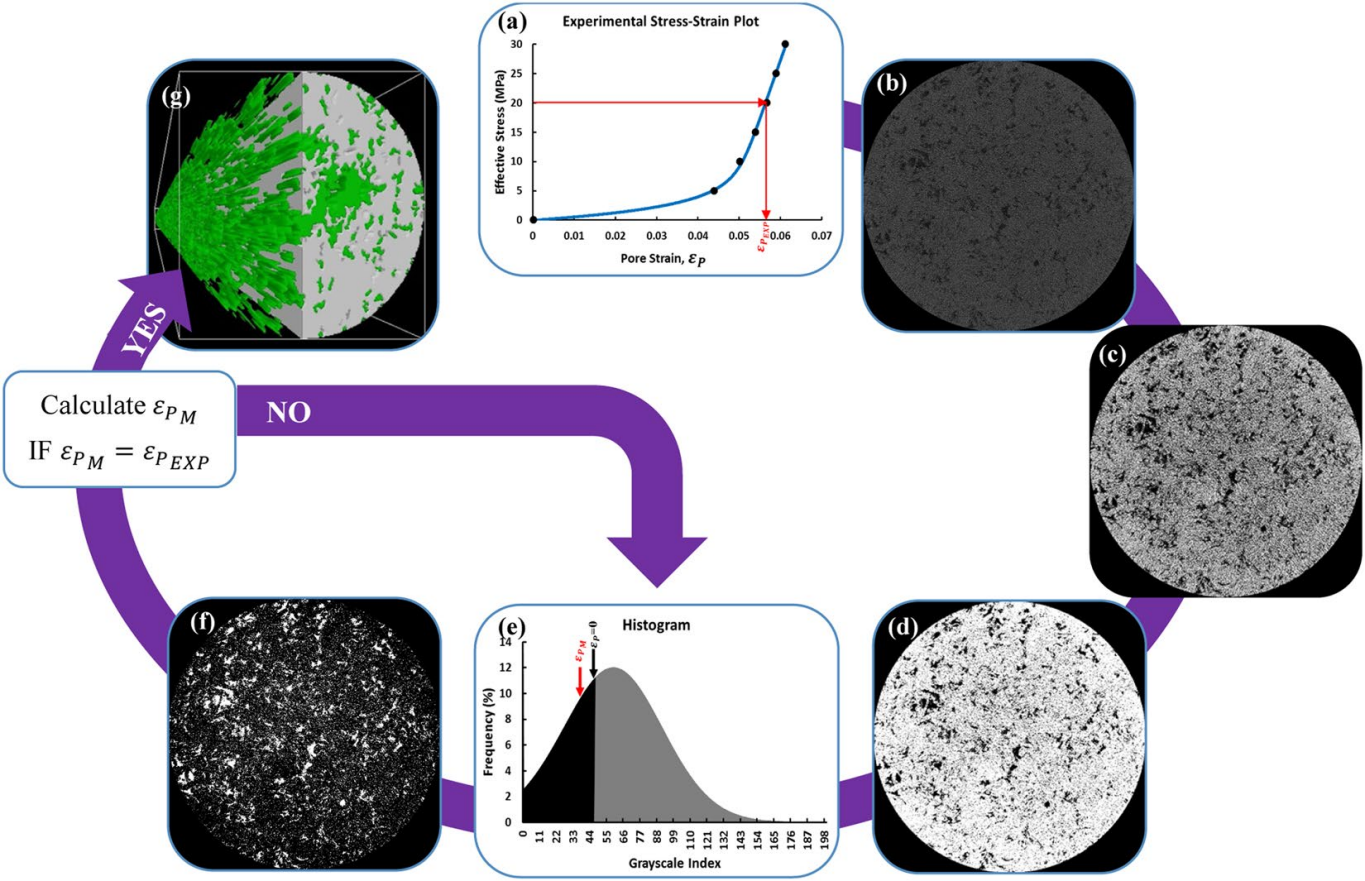
Proxy modelling work flow in 7 steps: (**a**) Choosing a new effective stress and find experimental pore strain ${\varepsilon }_{{p}_{EXP}}$ (**b**) Uploading original X-ray µCT Image (**c**) Filtering (**d**) Binarization (**e**) Thresholding (**f**) Bitwise operation (**g**) Proxy modelling and analysis based on model’s pore strain ${\varepsilon }_{{p}_{M}}$.

Before running the core-scale experiments, the carbonate specimen was scanned using a stationary X-ray µCT at atmospheric pressure condition. The resulting 3D pore-scale model was synthetically compacted to achieve a proxy structure for the compacted core that was compatible with the imposed effective confining stress conditions. Figure 1 shows the work flow of the process implemented here. More detailed explanations are provided in the *Materials and Methods*.

## Analytical Modelling of Stress-Dependent Multiphase Flow Properties

As noted in^46^, natural porous media are fractals, a fact which has led many researchers to adapt fractal scaling laws to describe the transport and other pore-related properties. Here, the fractal nature of pore sizes was used to develop analytical models for stress-dependent capillary pressure and relative permeability curves, allowing for a better understanding of flow mechanisms on the scale of pores. Based on the fractal scaling laws, the cumulative size-distribution (*N*($\bar{r}$)) of pores with diameter ($\bar{r}$) greater than or equal to *r* was assumed to follow the fractal scaling law given bellow^55^:

1 $$N(\bar{r}\ge r)={({r}_{max}/r)}^{{D}_{f}}$$

where *D*_*f*_ and *r*_*max*_ are the fractal dimension and maximum pore diameter in porous media, respectively. Based on this definition, the equation for stress-dependent capillary pressure *P*_*c*_(*σ*′) can be shown to be

2 $${P}_{c}(\sigma ^{\prime} )=(\frac{{P}_{{e}_{i}}}{\sqrt[3-{D}_{T}]{1-{\varepsilon }_{p}}}){{S}_{w}}^{-\frac{1}{\lambda }}.$$

The terms *P*_*e*_, *ε*_*p*_, *λ* = 3 − *D*_*T*_ − *D*_*f*_ and *D*_*T*_ in eq. (2) refer to entry capillary pressure, pore strain, stress-dependent pore size distribution index and tortuosity fractal dimension, respectively, and the index *i* indicates the initial stress condition (*σ*′ = 0) in this study. Based on the non-linear stress-strain equation for the poroelastic materials, *ε*_*p*_ is related to the changes in effective confining stress (*σ*′) using the following equation^56^, assuming pore strain (*ε*_*p*_) to be equal to the volumetric strain (*ε*_*v*_).

3 $${\varepsilon }_{p}=-\,\frac{d{V}_{p}}{{V}_{pi}}=A\sigma ^{\prime} -B{e}^{-\sigma ^{\prime} /C}+B$$

where A, B, and C are fitting constants. Haghi *et al*.^34^ defined the fitting constants in eq. (3) as *A* = (1 − *γ*_*s*_)/*K*_*H*_, *B* = *γ*_*s*_, and *C* = *K*_*S*_, where *K*_*H*_ and *K*_*S*_ are the bulk moduli of hard and soft parts of the rock, respectively. *γ*_*s*_ refers to a ratio of the soft part volume to the bulk volume at an unstressed condition. Mixing eq. (2) with Burdine’s empirical equation^19^, the following semi-analytical equations are developed for relative permeability curves *k*_*r*_(*σ*′).

4 $${k}_{rw}(\sigma ^{\prime} )={(\frac{{S}_{w}-{S}_{wir}}{1-{S}_{wir}})}^{2}\frac{{{S}_{w}}^{\frac{2+\lambda }{\lambda }}-{{S}_{wir}}^{\frac{2+\lambda }{\lambda }}}{1-{{S}_{wir}}^{\frac{2+\lambda }{\lambda }}}$$

5 $${k}_{rg}(\sigma ^{\prime} )={k}_{rg-max}{(\frac{1-{S}_{w}}{1-{S}_{wir}})}^{2}\frac{1-{{S}_{w}}^{\frac{2+\lambda }{\lambda }}}{1-{{S}_{wir}}^{\frac{2+\lambda }{\lambda }}}$$

where, *S*_*wir*_ and *k*_*rg−max*_ are irreducible water saturation and maximum gas relative permeability, respectively. A detailed derivation of the above equations is given in *SI Appendix A*.

## Results and Discussions

### Core-Scale Approaches

**Figure 2:**
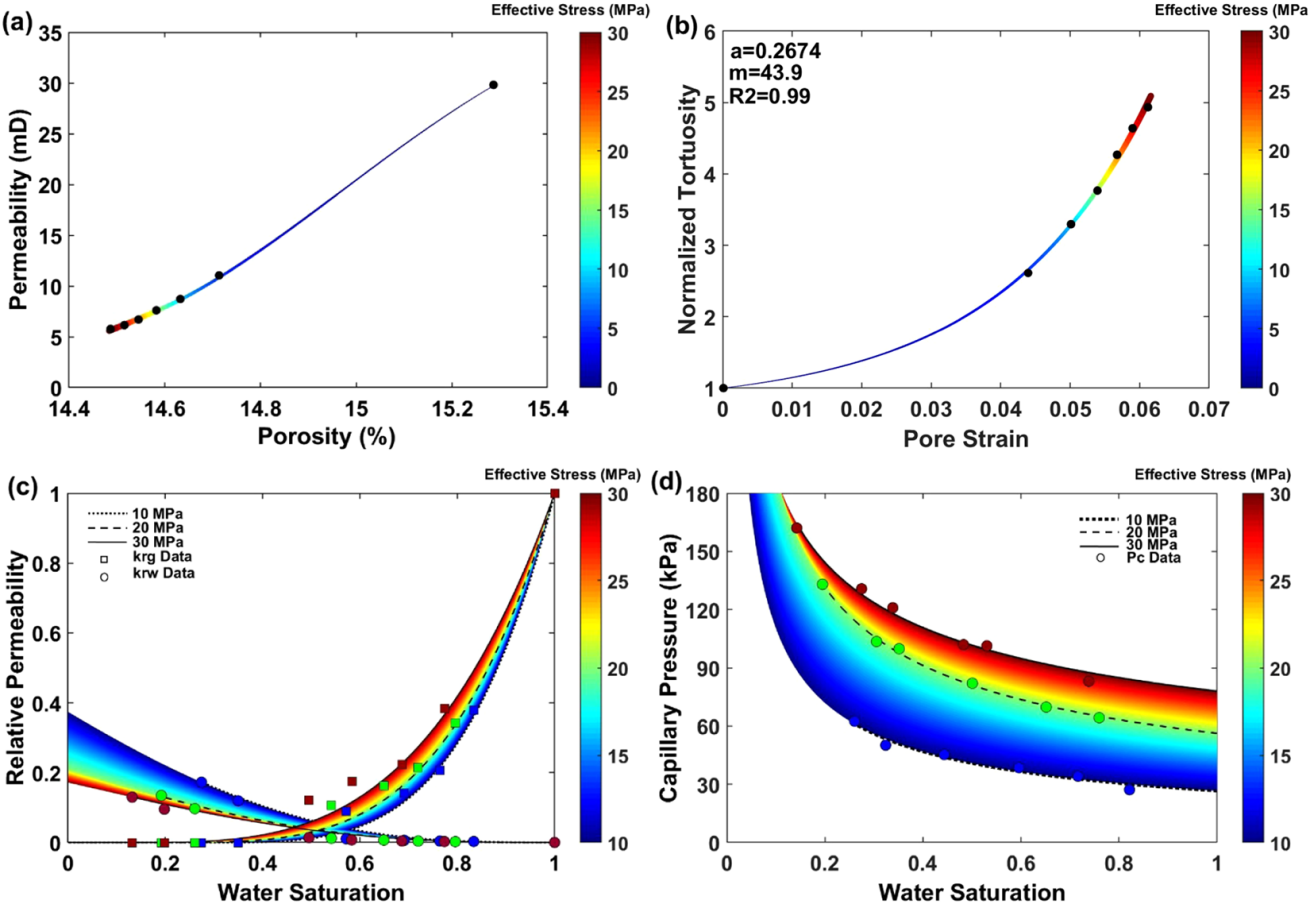
Core-scale experimental results representing the stress dependency of structural, single-phase flow, and multiphase flow properties of the carbonate rock: (**a**) stress-dependent permeability versus porosity plot, (**b**) normalized tortuosity versus pore strain, (**c**) stress-dependent relative permeability, and (**d**) stress-dependent capillary pressure.

The main contributions of the current research at the core-scale are the experimental results illustrated in Fig. 2 of the stress-dependent structural, single-phase flow, and multiphase flow properties of a porous carbonate rock under a wide range of isotropic effective confining stress conditions. In Fig. 2, circles and squares represent experimental data and colors indicate various effective confining stress conditions. Figure 2(a) shows the decline in stress-dependent permeability (*k*) versus stress-dependent porosity (*φ*) in response to an increasing effective stress condition, where the colored curve is fitted on the experimental data using the following equation based on the Carman and Kozeny correlation^57^.

6 $$k={k}_{i}{(\frac{\phi }{{\phi }_{i}})}^{3}{(\frac{1-{\phi }_{i}}{1-\phi })}^{2}(\frac{{\tau }_{i}}{\tau })$$

Here, *τ* defines the flow path hydraulic tortuosity inside the porous medium. The black circles in Fig. 2(b) represent the calculated normalized tortuosity ($\bar{\tau }=\tau /{\tau }_{i}$) from eq. (6) (at known stress-dependent *φ* and *k* points) and the experimental pore strain data of the sample. The fitted curve is plotted based on the following equation^34^,

7 $$\bar{\tau }=a({(1-{\varepsilon }_{P})}^{-m}-1)+1$$

where the grain shape factor (*a*) and material constant (*m*) are calculated using the least squares regression technique (Fig. 2(b)). This plot clearly reveals an increasing intensity of flow path tortuosity under increasing effective stress conditions. This confirms the simultaneous dual-effect of stress-induced deformation on both pore and connecting-channels sizes, where these are comprehensible through pore strain and hydraulic tortuosity variation, respectively.

Fig. 2(c,d) illustrate the core-scale experimental results of how the stress-dependent multiphase flow properties vary under a range of effective confining stress condition from 10 MPa to 30 MPa. These two plots are an experimental demonstration of the remarkable controls that geomechanical processes impose on the multiphase flow properties of a porous carbonate rock. Herein, eq. (2) and (4–5) are applied as the curve fitting and interpolating equations. More details are given in *SI Appendix A*.

The results illustrated in Fig. 2(c,d) show leftward (decreasing *S*_*wir*_) and upward shifts in the relative permeability and capillary pressure curves, respectively. These shifts are conceptually an indication of a decrease in pore sizes, which will be investigated in the next section.

**Figure 3:**
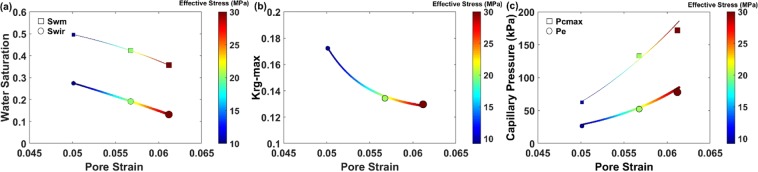
The plots provide stress-dependent (**a**) irreducible water saturation (*S*_*wir*_) and water saturation at the intersection point of water and gas relative permeability curves (*S*_*wm*_), (**b**) maximum gas relative permeability (${k}_{rg-max}$), and (**c**) entry and maximum capillary pressure (*P*_*e*_ and ${P}_{cmax}$, respectively) versus pore strain.

Intuitively, one would expect that a porous medium’s increasing affinity to the gas phase should be responsible for the leftward shift of the relative permeability curves and decrease in *S*_*wir*_. However, in our experiments, the interfacial tension and fluid properties were constant. To provide quantitative insight into the increasing affinity of the porous media to the gas phase, we provide important evidence from the experimental data at each stress condition: (1) irreducible water saturation (Fig. 3(a)), (2) water saturation at the intersection point of water and gas relative permeability curves (*S*_*wm*_, Fig. 3(a)), and (3) maximum gas relative permeability (Fig. 3(b)). These observations, together with our knowledge of the non-changing surface and interfacial tension between the phases and solid during the experiments, reveal the remarkable influence of pore and/or channel deformation on our understanding of the wettability of porous media. Figure 3(c) illustrates an increase in dynamic capillary pressure end-points as effective stress is increased at constant flow rate conditions. The classical capillary number, defined as the ratio of the viscous forces to the capillary forces (i.e. $Ca={\mu }_{g}{Q}_{inj}/\gamma A$)^58^, increases slightly as the core cross-sectional area (*A*) decreases in response to an increase in effective stress. For capillary number calculations, gas viscosity (${\mu }_{g}=0.184\,mP.s$), injection rate (${Q}_{inj}=8\,mL/min$), and interfacial tension ($\gamma =69.36\,mN/m$) are taken as stress-independent parameters. Mechanistically, the stress-induced increase of *Ca* (3.19–3.41 × 10^−8^) under increasing effective stress conditions (0–30 MPa) improves the likelihood of capillary desaturation of the water phase in the porous media, based on the capillary desaturation curve (CDC) for drainage process^58^; this is consistent with our experimental observations, in which *S*_*wir*_ declines as effective stress increases (Fig. 3(a)). However, given the limited range of calculated *Ca* for our experiments, in the next section further investigations are warranted for a more representative definition of the capillary number at the pore-scale with more reasonable water capillary desaturation values.

### Pore-Scale Approaches

In this section, pore-scale manifestations are evaluated to postulate physical mechanisms supporting the significant core-scale stress-dependent multiphase flow properties measured in our experiments. The proxy modeling procedure given in Fig. 1 is used to develop conceptual 3D models of deformation at the pore-scale.

**Figure 4:**
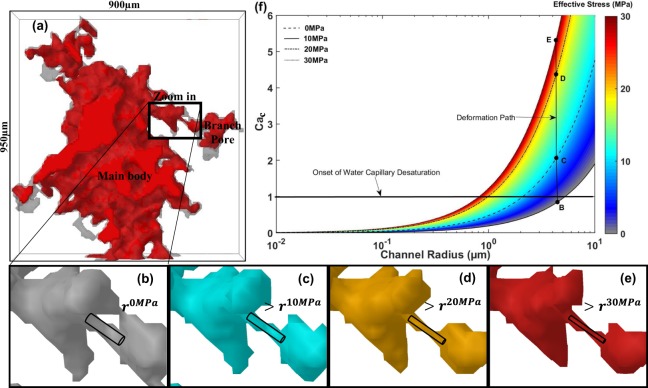
This figure presents: (**a**) a 3D image of a single pore in the carbonate core (gray) and its proxy model at 30 MPa effective stress condition (red); a single pore channel at (**b**) 0 MPa, (**c**) 10 MPa, (**d**) 20 MPa, and (**e**) 30 MPa effective confining stress condition; and (**f**) a 3D plot of the critical capillary number ($C{a}_{c}$) versus the channel radius at a range of effective stress conditions from zero to 30 MPa.

A qualitative demonstration of stress-induced capillary desaturation of the water phase in a single pore of our carbonate sample is presented in Fig. 4. Here, different colors indicate different effective stress conditions; the color bar corresponding to each is shown on the right side. 3D images of the selected pore at 0 MPa (gray) and 30 MPa (red) effective stress conditions are compared in the same frame in Fig. 4(a). The section in the solid black rectangle is magnified in Fig. 4(b–e) to depict the contraction of a channel connecting the main body to a branch pore with effective stress evolution from 0 MPa to 30 MPa.

Increasing effective stress, a simultaneous increase in both maximum applied capillary pressure (Fig. 3(c)) and channel capillary pressure occurs as the capillary radius *r* decreases (Fig. 4(b–e)) following the Young-Laplace equation ${P}_{c}=2\gamma \,\cos (\alpha )/r$^58^. When the applied core-scale capillary pressure (${P}_{c}^{Applied}={({P}_{g}-{P}_{w})|}_{sscond.}$) becomes equal to the channel capillary pressure at a critical *Ca*, the gas viscous pressure (Δ*P*_*g*_) becomes sufficient to expel, counter-currently, the water phase inside the pore through the channel. This leads to capillary desaturation of the water phase inside the pore. Gas viscous pressure is at its highest level at maximum applied capillary pressure (${P}_{cmax}^{Applied}$) under each effective stress condition where water saturation decreases to its minimum value at *S*_*wir*_. Scaling down the classical *Ca* definition for a pore-channel in a porous media, the terms viscous forces and capillary forces can be replaced with viscous pressure and capillary pressure of the channel, respectively^59^. At critical state, maximum gas viscous pressure (Δ*P*_*gmax*_) at maximum flow rate and steady-state condition, where water phase is immobile, is assumed to be equal to ${P}_{cmax}^{Applied}$^34,54^.

8 $$C{a}_{c}=\frac{\Delta {P}_{gmax}}{{P}_{c}}=\frac{{P}_{cmax}^{Applied}\times r}{2\gamma \,\cos (\alpha )}.$$

Equation (8) provides a new definition for the critical capillary number by connecting microscopic and macroscopic terms in a porous media to distinguish the onset of capillary desaturation of water at $C{a}_{c}=1$ during the constant flow gas drainage process. Using eq. (8), Fig. 4(f), which provides a multifunctional phase redistribution diagram under variable effective stress conditions, illustrates new insights for stress-dependent critical capillary numbers associated with the pore-channel radius of the carbonate specimen used in this study.

At each effective stress condition, the threshold radius of the medium’s channels, in which the capillary desaturation of water starts, can be obtained at $C{a}_{c}=1$. Additionally, the transition of a single channel, through its deformation path, from a gas capillary barrier to a gas flow conduit (or reverse) at different stress conditions can be recognized. With respect to the channel illustrated in Fig. 4(b–e), increasing effective stress from zero to 10 MPa (Fig. 4(b,c)) results in a transition in the state of the channel from a gas barrier to gas flow conduit; this leads to capillary desaturation of the water phase inside the branch pore. This microscopic transition explains the macroscopic decrease in *S*_*wm*_ and *S*_*wir*_ in response to an increase in effective stress (Fig. 3(a)). A decrease in *S*_*wm*_ and *S*_*wir*_ can be translated into an increasing affinity of porous media for the gas phase by increasing effective stress.

**Figure 5:**
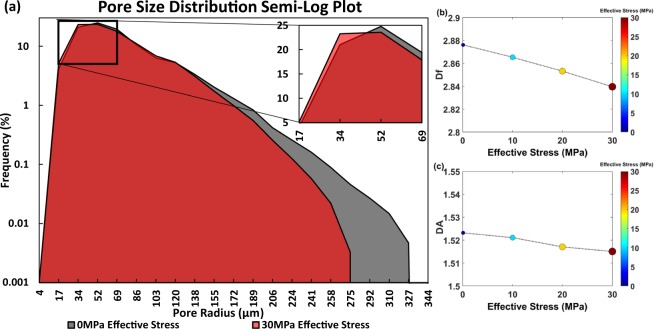
This figure provides insights into stress-dependent (**a**) pore size distribution in a semi-log plot, (**b**) 3D fractal dimension (*D*_*f*_), and (**c**) 3D degree of anisotropy (*D*_*A*_) at a range of effective stress conditions from zero to 30 MPa for the carbonate sample.

To provide further quantitative description of stress-dependent changes in pore-scale structure, pore size distribution, with pore radius (*r*) being ≥4 *μm* (Fig. 5(a)), and two metrics, namely 1) 3D fractal dimension (*D*_*f*_) which quantifies how 3D objects fills pore spaces (Fig. 5(b)) and 2) degree of anisotropy (*D*_*A*_), which is a measure of 3D symmetry of pores in the media (Fig. 5(c)), are calculated at each effective stress condition. Details on these calculations are given in the *Materials and Methods*.

As a result of increasing the effective confining stress from 0 MPa to 30 MPa, a leftward shift in effective pore size distribution (PSD) is observed (Fig. 5(a)), while the mean pore radius decreases from 70 *μm* to 67 *μm*, respectively. Additionally, the cumulative frequency of pores with *r* ≤ 52 *μm* increases from 49% at 0 MPa effective stress to 52% at 30 MPa effective stress; the remaining pores span up to 326.85 *μm* and 275.25 *μm*, respectively. These stress-dependent declines in the mean pore radius and size of the largest effective pore, pore connected to the surface of the core, result in an increasing trend of capillary pressure curves and entry capillary pressure, respectively^58^, in response to an increase in effective stress. A similar trend can be realized at the core-scale using the stress-dependent capillary pressure experimental results shown in Fig. 2(d). Additionally, the leftward shift in PSD justifies the decline in maximum gas relative permeability under increasing effective stress conditions (Fig. 2(b)).

A declining trend was observed in both dimensionless numbers *D*_*f*_ and *D*_*A*_ in response to an increase in effective stress from 0 MPa to 30 MPa (Fig. 5(b,c)). These two pore structural metrics quantify clearly the rock deformation and its impacts on single-phase and multiphase parameters. The decreasing trend of *D*_*f*_ is an indication of more uniform (i.e. well-sorted) pore sizes^58^; its striking impact on decreasing porosity, absolute permeability, and *S*_*wir*_ and increasing slope of capillary pressure curves has been shown experimentally and analytically in the literature^24,46–50^. In a similar way, our calculated *D*_*A*_ represents an increasing state of isotropy of the sample due to pore compaction in response to increasing effective stress.

## Implicational Insights

Simultaneous flow of gases and liquids in natural porous media is an indispensable phenomenon to study when we are dealing with subsurface flow processes. Stress-dependent shifts in the relative permeability and capillary pressure curves have a major impact on our classical methods of understanding, quantifying, and modeling multiphase fluid flow in subsurface formations for a wide range of engineering and natural applications, including enhanced oil recovery, CO_2_ subsurface storage, heat energy recovery from geothermal reservoirs, and surface water infiltration in the vadose zone.

Although in this study we have conducted the flooding experiments on a standard carbonate specimen using water (wetting phase) and N_2_ (non-wetting phase) phases, the discovered systematic stress-dependent behavior of rock could be extended for a wider range of rocks such as sandstone^34^ and fluid pairs (e.g. gas + liquid), as gases are typically the non-wetting phase for most natural porous media. For instance, the stress-dependent upward shift in the entry capillary pressure for the studied carbonate rock, at an increasing effective stress condition from 10 MPa to 30 MPa (Fig. 3(c)), almost doubles the injection pressure and energy required to push the gas phase into the porous rock. However, the stress-dependent decrease in irreducible wetting phase saturation resulting from increasing the effective stress condition from 10 MPa to 30 MPa (Fig. 3(a)) leads to an almost 20% increase in the volume of stored gas inside the core and recovery of the wetting phase (i.e. water) from the core. These dramatic changes in gas injection pressure, volume of stored gas, and recovery of the wetting phase are technically and economically significant for similar scenarios such as CO_2_ subsurface storage and enhanced recovery of hydrocarbon reservoirs using an miscible/immiscible gas (e.g. CO_2_) flooding technique. These stress-dependent processes allow us to predict additional CO_2_ storage in subsurface formations. In the same way, stress-dependent leftward shifts in the relative permeability curves facilitate rainwater infiltration into the vadose zone and hot water flow through two-phase geothermal reservoirs. This fundamental study paves the way for a more realistic study of fluid flow mechanisms in any natural and industrial applications which are dealing with multiphase fluid flow properties of rock under variable effective stress conditions.

## Summary and Conclusions

We have shown experimentally the systematic impact of pore deformation as a result of effective stress changes on the single-phase and multiphase fluid flow properties of a carbonate specimen via a series of core-flooding experiments using water/N_2_ phases under isothermal and triaxial isotropic effective stress conditions. Our experiments have revealed leftward and upward shifts in the relative permeability and capillary pressure curves, respectively, with increasing effective stress, and have shown a decreasing shift of *S*_*wir*_, *S*_*wm*_, and *k*_*rwmax*_ under increasing effective stress conditions. These experiments have provided us core-scale insights into the linkage between these shifts to decreases in porosity and absolute permeability and increases in pore strain and pore flow tortuosity under an increasing effective stress condition. Fractal and poroelasticity theories were used to drive analytical equations for stress-dependent relative permeability and capillary pressure curves. These equations were used for curve fitting and interpolation in this study.

Using X-ray computed micro-tomography and a proxy modelling technique, we were able to quantify the structure and shape of the pores and channels, pore size distribution, and scaling dimensionless numbers (e.g. fractal dimension and degree of anisotropy) at different effective stress conditions. We have shown that increasing effective stress leads to a leftward shift in PSD and closure of micro-channels. As revealed in the 3D phase redistribution diagram (Fig. 4(f)), we have quantified the stress-dependent threshold radius of the medium’s channels at the starting point of capillary desaturation of water in the porous media. Additionally, we have revealed the transition of a single channel, through its deformation path, from a gas capillary barrier into a gas flow conduit in response to an increasing stress conditions, which resulted in the capillary desaturation of the water phase and a macroscopic decrease in *S*_*wm*_ and *S*_*wir*_. We have further revealed that leftward shift in PSD is responsible for the increasing trend of capillary pressure curves under an increasing effective stress condition. *D*_*f*_ and *D*_*A*_ metrics proved quantitatively the role of effective stress condition on pore size distribution and pore isotropy, respectively, in porous media.

These findings underscore the significant impact of effective stress on multiphase flow properties of rock, and manifest the physical mechanisms, such as capillary desaturation and capillary gas blockage due to stress-induced mechanical deformation of pores and/or channels, that control those properties.

## Materials and Methods

### Core-Scale Experiments

The experiments were conducted on a water-wet Indiana limestone specimen (from Kocurek Industries INC., USA) 3.81 cm in diameter and 10.16 cm in length. The uniaxial compressive strength and Young’s modulus of the rock were 25.78 MPa and ≈3 GPa, respectively (Fig. S1). N_2_ and deionized water were used as the flooding phases and CO_2_ as the displacing phase. Here, an air-water contact angle (*α*) of 18.8 degrees were measured using a drop shape analyzer (DSA), which indicated the strong water-wet nature of the specimen (Fig. S2). Full re-saturation of the confined sample inside the cell was a key challenge that we tackled using a procedure consisting of 3 steps: 1) flushing the core with 50 pore volume of high pressure CO_2_, 2) vacuuming the sample (at ambient water saturation pressure) from an outlet line over a liquid nitrogen cold trap until a plateau pressure was achieved at the inlet, and 3) injecting high pressure water into the sample and maintaining the pressure for a few hours before re-flushing it with 50 pore volume of water. The experimental procedure in this study is analogous to the protocol described in ref.^34^ for Berea sandstone, to which readers are referred for additional details.

### Imaging and Proxy Modelling

The X-ray micro-tomography was performed using the micro-CT imaging suite in the Pharmaceutical Orthopedic Research Lab (PORL) at the University of Alberta. The X-ray source within this equipment is a sealed tube with a voltage of 100 kV and a spot size of <5 µm. The imager scans 360 degrees rotational field-of-view at a time using a 12 bit X-ray detector that is fiber-optically coupled to a scintillator. We scanned an 18 mm length of the full-diameter core 5 mm away from the end face of the core. All tomographic images were at 8.6 μm per voxel resolution. The post-processing and quantifications were performed using the CT Analyser program within Bruker microCT 3D Suite software. The proxy modeling process in this study consisted of 7 steps, and the pore stress-strain plot of the rock from the core-scale experiments was an initial input in this cycle. In the first step, we chose an effective stress condition and identified the corresponding pore strain from our core-scale experiments (${\varepsilon }_{{p}_{EXP}}$). Then the original X-ray µCT images were filtered using median and contrast enhancement techniques. Filtered images were segmented into black and white binary areas representing pores and grains, respectively. Through the thresholding step, a grayscale index smaller than its original value for the rock at zero effective stress was selected manually. Otsu’s thresholding method^60^ in 3D space was used initially to define the original grayscale index and match the model’s 3D porosity with experimentally measured porosity at zero effective stress. The model’s pore system was then assigned as a 3D object through bitwise operation, and its volume and strain (${\varepsilon }_{{p}_{M}}=({V}_{{p}_{Mi}}-{V}_{{p}_{M}})/{V}_{{p}_{Mi}}$) were calculated. ${V}_{{P}_{Mi}}$ and ${V}_{{P}_{M}}$ defined as proxy model’s initial and stress-dependent pore volume, respectively. This process was repeated iteratively until the pore strain of the proxy model became equal (±0.1%) to its corresponding experimental value and the representative model used for further 3D analysis.

### Pore Size Distribution and Pore Metrics

We implemented the 3D calculation of pore size distribution, fractal dimension, and degree of anisotropy using the CT Analyser program within Bruker microCT 3D Suite Software. For the pore network process, firstly, a “skeletonisation” was performed to identify the medial axis of all pores. Secondly, a “sphere-fitting” measurement was made for all of the voxels along this axis. Pore size is defined as the diameter of the largest sphere which meets two conditions: (1) the sphere encloses the point (but the point is not necessarily the center of the sphere), and (2) the sphere is entirely located within the pore. The Kolmogorov or box-counting method was used to calculate *D*_*f*_ in 3D; following this process, the volume was divided into an array of equal cubes and the number of cubes containing part of the object was counted. The process was repeated over a range of box sizes (3 to 100 pixels). Plotting the box sizes (L) versus the number of occupied boxes (*N*(*L*)), the slope of log-log linear regression defined *D*_*f*_. Note that a linear relationship between the number of occupied boxes and box sizes in a log-log plot with the slope of *D*_*f*_ is also a confirmation of fractal nature of the porous sample based on $N(L)\propto {L}^{-{D}_{f}}$, where this was the case in the current study with the slopes given in Fig. 5(b). Finally, mean intercept length (MIL) analysis was used to measure *D*_*A*_. As part of this process, a grid of lines was sent through the 3D core image containing pores (object) and grains (void) over a large number of 3D angles, and the length of the test line through the analyzed volume was divided by the number of times that the line passed through or intercepted pores. For more details, readers are referred to the software manual (https://www.bruker.com/products/microtomography.html).

## Supplementary information

Supplementary
