# Supplementary material for: Stress-Dependent Pore Deformation Effects on Multiphase Flow Properties of Porous Media
Source: Sci Rep. 2019 Oct 18;9:15004. doi: 10.1038/s41598-019-51263-0 (PMC6802081; doi:10.1038/s41598-019-51263-0)
Supplement: Supplementary file 1 — Supplementary [file 41598_2019_51263_MOESM1_ESM.pdf]

Supplementary Information for

Stress-Dependent Pore Deformation Effects on Multiphase Flow Properties of Porous Media

Amir H. Haghi [1,*], Richard Chalaturnyk [1], and Stephen Talman [1]

[1]Reservoir Geomechanics Research Group, University of Alberta, Edmonton, T6G 1H9, Canada.

[*] haghi@ualberta.ca

This PDF file includes:

Appendix A
Figures S1 and S2

Appendix A

Analytical Model Derivation

Here, we describe the mathematical process to develop Eq. (2) and Eqs. (4)-(5) based on the fractal scaling law (N) given in Eq. (1) and its first derivative ($dN = -D_f r_{max}^{D_f} r^{-D_f-1} dr$). To model the classical capillary pressure plots (e.g. Fig. 2(d)) at each certain water saturation (S_w) with the application of fractal capillary tubes, we employ a typical simplifying assumption dividing the tubes into two groups: 1) tubes with a radius smaller than r, which are fully filled with water and 2) the remaining tubes which are fully filled with gas; we ignore the volume of the thin film of water with a thickness on the order of nanometers. On the basis of this assumption, S_w in the porous media is calculated using the following equation (50):

$$S_w = \frac{V_{water}}{V_p} = \frac{\int_{r_{min}}^{r} \pi r^2 L_r dN}{\int_{r_{min}}^{r_{max}} \pi r^2 L_r dN}, \quad (S1)$$

where the actual length of the convoluted capillary tubes (L_r) is assumed to follow the fractal scaling law $L_r = r^{1-D_T} L^{D_T}$ (55). Here, D_T and L are the tortuosity fractal dimension and representative length of straight capillary tubes, respectively, where at D_T equal to 1 (straight tubes), $L_r = L$. Integrating Eq. (S1), we derive the following relationship for S_w,

$$S_w = \left(\frac{2\gamma\cos(\alpha)}{r_{max} P_c}\right)^{3-D_T-D_f}. \quad (S2)$$

In Eq. (S2), we replace the tube radius r with P_c using the Young-Laplace equation (59) and assume the fraction of minimum to maximum tube radius (r_{min}/r_{max}) in the medium is negligible, which is a reasonable assumption for most natural porous rocks (55). Under a homogeneous pore strain condition, the strain of the tube with maximum radius (r_{max}) is derived as follows:

$$\varepsilon_p = 1 - \frac{V_p}{V_{pi}} = 1 - \left(\frac{r_{max}}{r_{maxi}}\right)^{3-D_T}. \quad (S3)$$

For simplification, D_T and L for the tube with maximum radius are assumed here to be stress-independent, although the actual length (L_r) remains stress-dependent. Mixing Eq. (S2) with Eq. (S3) and rearranging it, by applying the Young-Laplace equation (59) to the tube with maximum radius ($P_e = 2\gamma\cos(\alpha)/r_{max}$), leads to Eq. (2) for stress-dependent capillary pressure curve as a function of water saturation, which is analogous to the well-known Brooks and Corey empirical equation (20). The first term on the right-hand side of Eq. (2) defines the stress-dependent entry capillary pressure,

$$P_e(\sigma') = \frac{P_{e_i}}{\sqrt[3-D_T]{1-\varepsilon_p}}. \quad (S4)$$

For the final step, we replace the P_c term in the Burdine empirical equations (19) for wetting (water) and non-wetting phase (gas) drainage relative permeability curves (Eq. (S5) and (S6), respectively) with the derived definition of P_c in Eq. (2) and integrate Eqs. (S5)-(S6) to develop Eqs. (4)-(5) for $k_{rw}(\sigma')$ and $k_{rg}(\sigma')$, respectively.

$$k_{rw} = \left(\frac{S_w - S_{wir}}{1 - S_{wir}}\right)^2 \frac{\int_{S_{wir}}^{S_w} dS_w/(P_c)^2}{\int_{S_{wir}}^{1} dS_w/(P_c)^2} \quad (S5)$$

$$k_{rw} = k_{rg-max} \left(\frac{1 - S_w}{1 - S_{wir}}\right)^2 \frac{\int_{S_w}^{1} dS_w/(P_c)^2}{\int_{S_{wir}}^{1} dS_w/(P_c)^2} \quad (S6)$$

Analytical Model Results.

We use power-law fitting correlation ($ax^b + c$) to interpolate the stress-dependent S_{wm}, S_{wir}, and k_{rg-max} data as a function of pore strain in Figs. 3(a)-(b). Fitting the non-linear stress-strain correlation equation (Eq. (3)) to the experimental data (Fig. 1(a)), we measure the constants γ_S, K_H, and K_S as being equal to 0.046, 1.91 GPa, and 2.328 MPa, respectively. Knowing the stress-strain relationship, we quantify $P_e(\sigma')$

using Eq. (S4) with the fitting constants $D_T = 2.99$ and $P_{e_i} = 0.3kPa$; the curve is illustrated in Fig. 3(c). Then, fitting the derived formula for stress-dependent capillary pressure (Eq. (2)) on the experimental data with the application of least squares regression technique, we estimate stress-dependent λ to be equal to 1.62, 1.9, and 2.612 at 10, 20, and 30 MPa effective confining stress conditions, respectively. In the same way, we use power-law fitting correlation ($\lambda = a(\sigma')^b + c$) to interpolate λ as a function of effective stress using the least square regression method. All of the fitting constants are summarized in Table S1. These interpolated stress-dependent properties are applied to Eq. (2) to model the stress-dependent capillary pressure data (Fig. 2(d)) and in the same method to Eqs. (4)-(5) to model the stress-dependent relative permeability data (Fig. 2(c)).

Table S1. Calculated fitting constants for the power-law correlation functions using least square regression technique.

**Parameter**	**Fitting Constant**		
	$\boldsymbol{a}$	$\boldsymbol{b}$	$\boldsymbol{c}$
S_{wm}	-3.322×10^4	4.231	0.6011
S_{wir}	-73.81	1.81	0.6023
k_{rg-max}	3.56×10^-16	-10.88	0.123
λ	5.384×10^-5	2.901	1.575

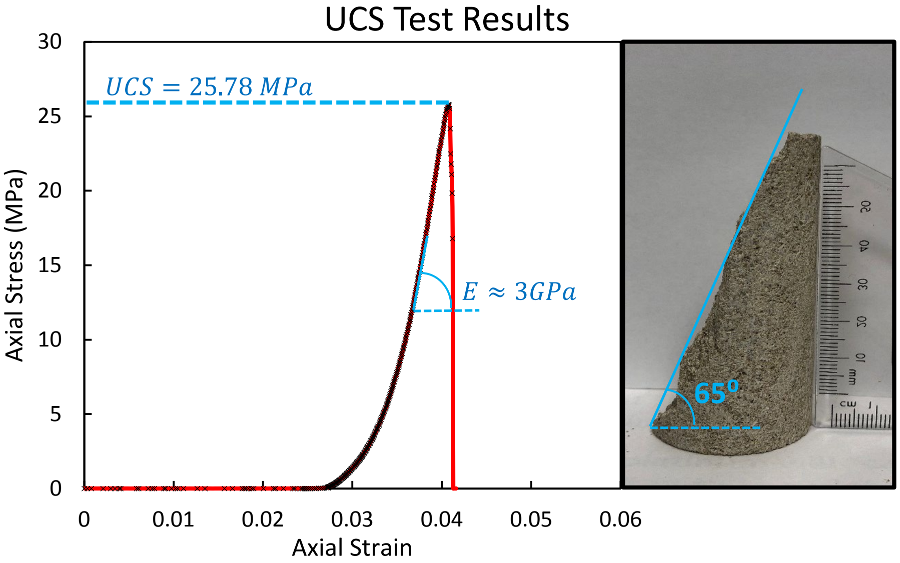

Figure S1. Uniaxial compressive strength test results on an Indiana limestone specimen (3.81cm in diameter and 7.62cm in length) measured based on ISRM suggested standard methods for determining the uniaxial compressive strength and deformability of rock materials (1). Based on the measured mechanical property of the core (UCS=25.78MPa), we classified it as a moderately hard rock as defined by NRCS-NEH (2).

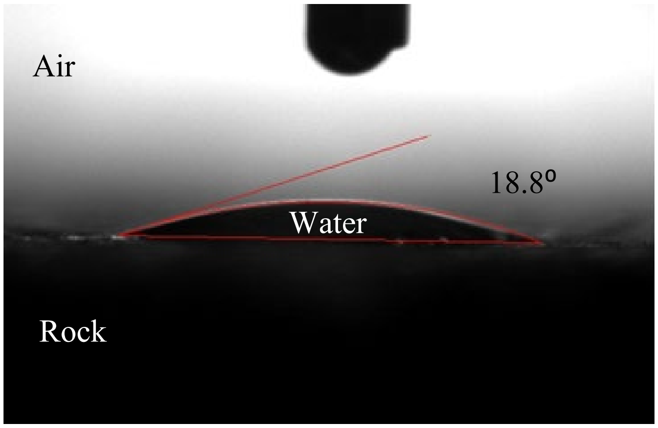

Figure S2. Measured air-water contact angle ($\boldsymbol{\alpha}$) equal to 18.8 degrees using a drop shape analyzer (DSA) at equilibrium condition. Based on the measured contact angle smaller than 90^0, the carbonate is categorized as a strong water-wet rock.

References

1. ASTM D7012, Standard Test Methods for Compressive Strength and Elastic Moduli of Intact Rock Core Specimens under Varying States of Stress and Temperatures
2. NRCS National Engineering Handbook (NEH), Part 631-Geology, Chapter 4- Engineering Classification of Rock Materials, 631.0401 Rock material properties, Section (c) (2012).
